# Genome-Wide Identification, Phylogenetic and Expression Analysis of Expansin Gene Family in *Medicago sativa* L.

**DOI:** 10.3390/ijms25094700

**Published:** 2024-04-25

**Authors:** Yajing Li, Yangyang Zhang, Jing Cui, Xue Wang, Mingna Li, Lili Zhang, Junmei Kang

**Affiliations:** 1 Institute of Animal Science, Chinese Academy of Agricultural Sciences, Beijing 100193, China; ly_yajing@yeah.net (Y.L.); yang20170115@163.com (Y.Z.); cuijing0417@yeah.net (J.C.); wangxue01@caas.cn (X.W.); limingna@caas.cn (M.L.); lili_chang721@yeah.net (L.Z.); 2 College of Grassland Agriculture, Northwest A&F University, Yangling 712100, China

**Keywords:** alfalfa, *Expansins*, genome-wide identification, abiotic stresses

## Abstract

Expansins, a class of cell-wall-loosening proteins that regulate plant growth and stress resistance, have been studied in a variety of plant species. However, little is known about the *Expansins* present in alfalfa (*Medicago sativa* L.) due to the complexity of its tetraploidy. Based on the alfalfa (cultivar “XinjiangDaye”) reference genome, we identified 168 *Expansin* members (*MsEXPs*). Phylogenetic analysis showed that MsEXPs consist of four subfamilies: MsEXPAs (123), MsEXPBs (25), MsEXLAs (2), and MsEXLBs (18). MsEXPAs, which account for 73.2% of MsEXPs, and are divided into twelve groups (EXPA-I–EXPA-XII). Of these, EXPA-XI members are specific to *Medicago trunctula* and alfalfa. Gene composition analysis revealed that the members of each individual subfamily shared a similar structure. Interestingly, about 56.3% of the *cis*-acting elements were predicted to be associated with abiotic stress, and the majority were MYB- and MYC-binding motifs, accounting for 33.9% and 36.0%, respectively. Our short-term treatment (≤24 h) with NaCl (200 mM) or PEG (polyethylene glycol, 15%) showed that the transcriptional levels of 12 *MsEXPs* in seedlings were significantly altered at the tested time point(s), indicating that *MsEXPs* are osmotic-responsive. These findings imply the potential functions of *MsEXPs* in alfalfa adaptation to high salinity and/or drought. Future studies on *MsEXP* expression profiles under long-term (>24 h) stress treatment would provide valuable information on their involvement in the response of alfalfa to abiotic stress.

## 1. Introduction

Alfalfa (*Medicago sativa* L.), a perennial legume referred to as “Queen of the Forages”, is one of the most widely planted forages in the world due to its regenerative ability and environmental sustainability. As a valuable source of plant protein and vitamins, the palatability of the forage makes it a favorable fodder for livestock. In countries with limited farmland for the cultivation of alfalfa, improving resistance to adverse environmental cues has become a major bottleneck for alfalfa production. 

Plant cell walls, composed of cellulose microfibers and matrix polysaccharides, are one of the most significant characteristics that distinguish plants from animals [1]. The cell wall is a very important structural material in plants, and regulates their growth and development, hormone regulation, and biotic and abiotic stress responses [2,3]. When subjected to environmental stresses, plant cell walls form a thin, tenacious, and fluid fiber layer that binds the cells together, which determines their shape, maintains the necessary mechanical strength and rigidity of plant tissue, and forms the first barrier against abiotic factors [1,4,5,6,7,8]. Cell-wall-loosening proteins (Expansins), which show non-enzymatic activity in the cell wall, play an important role in cell wall dilation by ensuring the normal growth and development of plants and responding to biotic and abiotic stresses in a variety of plant species [9]. Hence, exploring the response of alfalfa *EXPs* to high-salinity or drought conditions may provide information on the role of forage *MsEXPs* in the tolerance of adverse environments. 

The discovery of Expansin proteins was reported in Cosgrove’s study on the acid-induced elongation of cucumber (*Cucumis sativus* L.) hypocotyl cell walls in 1989 [10]. In 1992, McQueen Mason first isolated a protein from cucumber that possessed non-enzymatic activity and depended on pH to relax the cell walls of plants, and named it Expansin [11,12]. Expansins are a multigene family divided into four subfamilies: α-Expansin (EXPA), β-Expansin (EXPB), Expansin-like A (EXLA), and Expansin-like B (EXLB) [13,14]. Typical Expansins contain 250–275 amino acids in relatively conserved sequences, with a similarity of 20–40% [15,16]. Expansins have two domains, with domain 1 (N-terminal) being a double-psi β-barrel (DPBB), and domain 2 (C-terminal) sharing 50% similarity with Group-II pollen allergens [15,16,17]. The former cysteine-rich domain is generally considered to be a catalytic domain [16]. A signal peptide of 20–30 amino acid residues is present at the N-terminus [18]. In these subfamilies, EXPAs and EXPBs can loosen cell walls [13], while EXLAs and EXLBs are related to root architecture, hypocotyl length, and stress response [19,20]. The Expansin family has been widely studied in plant species such as mosses (*Physcomitrella patens* [21,22]), early vascular plants (*Selaginella moellendorffii* [23]), gymnosperms (*Ginkgo biloba* L. [24] and *Populus trichocarpa* [25]), monocotyledonous plants (*Oryza sativa* L. [26], *Triticum aestivum* L. [15], *Saccharum* spp. [27]), and dicotyledonous plants (*Arabidopsis thaliana* [17] and *Glycine max* L. [28,29]). The numbers of Expansin gene family members vary, which may be due to their methods of gene replication, including tandem replication and segmental replication [30]. Previous studies have shown that *Expansins* are involved in almost all physiological processes related to cell wall relaxation, including plant growth and development [31,32,33,34], seed germination [35], pollen tube elongation [14,36], and mycorrhizal formation [37]. In addition, *Expansin* is also involved in the nitrogen fixation process of legumes [38] and plant response to phytohormones [24,37] and abiotic stress [39,40,41,42,43,44]. For example, the overexpression of *NtEXPA11* enhanced the stress resistance of transgenic tobacco (*Nicotiana tabacum* L.) [42]. In addition, *RhEXPA4* may be related to the dehydration of roses (*Rosa hybrida*), and its expression is regulated by the transcription factor *RhNAC2* [41]. The *RhEXPA4*-overexpressing *Arabidopsis* lines displayed drought- and salt-tolerance characteristics. *Arabidopsis EXPA3*, *EXPA4*, *EXPA8*, *EXPA10*, and *EXLB1* have all been proven to be associated with drought adaptation [45]. The overexpression of wheat *TaEXPB23* enhanced drought and oxidative stress tolerance [39,43,44]. In *Brassica rapa* L., the expression level of *BrEXLB1* was positively correlated with drought tolerance and photosynthesis during the vegetative growth period [40]. These findings indicate the potential of *Expansins* in improving plant resilience to adverse environmental conditions. 

This study systematically analyzed the *Expansins* in the “XinjiangDaye” genome of alfalfa using whole-genome identification and conducted a comprehensive bioinformatics analysis of *MsEXPs*. Additionally, 12 *MsEXPs* were randomly selected, and their transcriptional expression levels were monitored under two environmental cues (salt and drought stress). This study provides a potential reference for understanding the characteristics of the alfalfa Expansin family and the molecular mechanisms of their response to salt and drought stress. 

## 2. Results

### 2.1. Phylogenetic Analysis of the Expansin Family Members in Alfalfa

**Figure 1:**
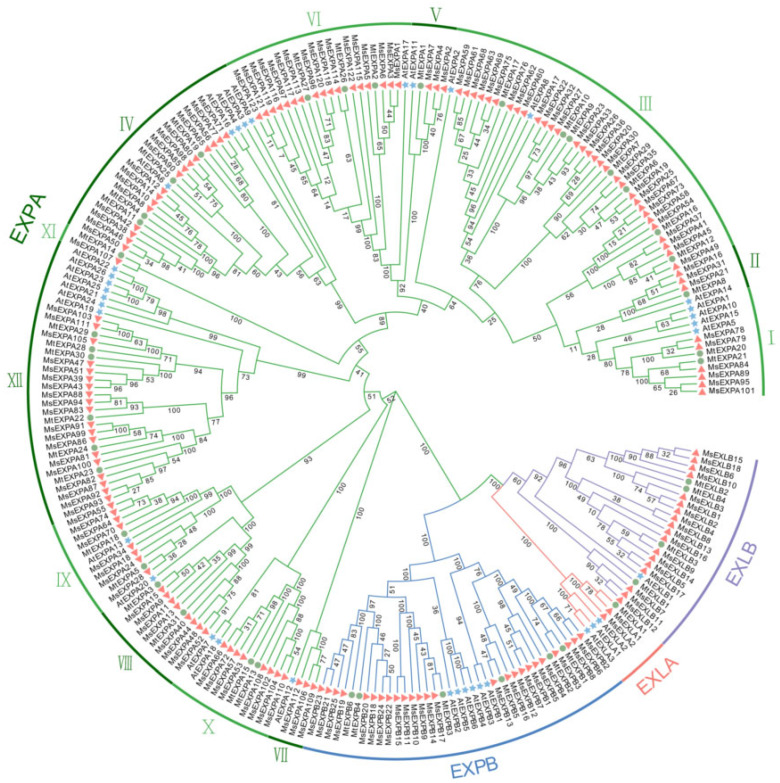
Phylogenetic tree of Expansin proteins. The unrooted phylogenetic tree was constructed using all Expansin proteins from alfalfa, *Medicago trunctula*, and *Arabidopsis thaliana* using the MEGA7.0 neighbor-joining (NJ) method with a bootstrap of 1000. Clades in green, blue, tangerine, and purple branches refer to the EXPA, EXPB, EXLA, and EXLB subfamilies, respectively. EXPA is divided into 12 subgroups (EXPAI–XII) and the subgroups are alternately marked in dark or light green. The tangerine triangles, green dots, and blue stars represent EXPs from alfalfa, *M. truncatula*, and *Arabidopsis*, respectively.

Expansin proteins are key determinants in plant cell-wall extension [46,47]. *Expansin*-mediated cell-wall loosening can affect tolerance of environmental stresses, such as drought [48,49], salt [50,51], and low-temperature [52,53] stress. Based on the Expansin proteins (EXPs) from *Arabidopsis*, the alfalfa (cultivar “XinjiangDaye”) genome [54] encodes 168 MsEXPs (Table S1). Phylogenetic analysis showed that MsEXPs, together with their orthologs from *Medicago trunctula* (42 MtEXPs) and *Arabidopsis* (36 AtEXPs), were clustered into four subfamilies, α-Expansin (EXPA, 123), β-Expansin (EXPB, 25), Expansin-like A (EXLA, 2), and Expansin-like B (EXLB, 18), according to the nomenclature of the AtEXPs [55] (Figure 1). Of these, the EXPAs were grouped into 12 subclusters (EXPA-I, -II, … -XII). Similarly to EXPs in barrel clover and *Arabidopsis*, MsEXPAs and MsEXPBs represented the majority of MsEXPs, accounting for about 73.2% and 14.9% of the EXPs in alfalfa, respectively. Relative to *Arabidopsis*, both *Medicago* species showed greater EXLBs and fewer EXLAs (Table S2). Among the 12 groups of EXPAs (EXPA-I–XII), EXPA-XI was specific to the two legumes, and each contained a single member (MtEXPA14 or MsEXPA107). 

### 2.2. The Features and Distributions of MsEXPs

The common features that MsEXPs share include two conservative domains: Double-psi beta-barrel Domain-1 (DPBB_1) and Pollen_allerg_1. The putative MsEXPs vary from 183 to 744 residues, with 95.8% (161/168) possessing about 200–300 amino acids (a.a.) (Table S1). Their molecular weight (MW) ranges from 20.78 kDa to 83.82 kDa, with 97.0% (163/168) showing values around 20 kDa–35 kDa. The isoelectric point (pI) of MsEXPs ranges from 4.52 to 9.98, with 82.5% (137/168) being alkaline and the rest being acidic. The prediction of subcellular localization revealed that 98.8% of the MsEXPs (166/168) were localized at the cell wall, with the exception of MsEXPA4 and MsEXPA7, which were predicted to be in the nucleus (Table S1). 

**Figure 2:**
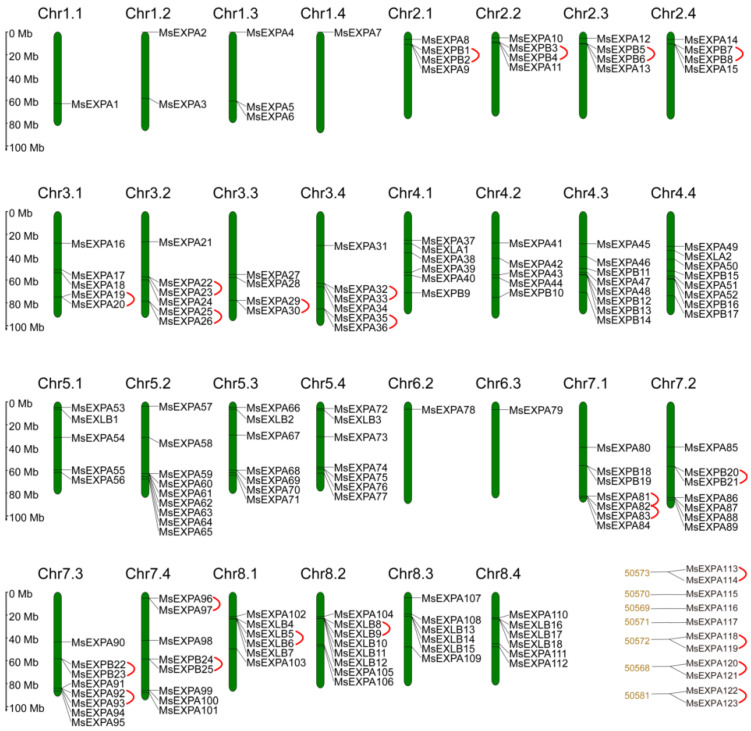
Chromosomal distribution of *MsEXPs*. Chromosome distributions of *MsEXPs* were visualized through Tbtools software v1.108 based on the physical location of each gene. The green vertical bars represent the chromosomes of alfalfa and are numbered on the top; 50,568–50,573 and 50,581 represent unplaced genomic scaffolds. Chromosome size is indicated by relative length. The scale (Mb) of chromosome length is displayed on the left. A total of 157 *MsEXPs* were mapped onto the 30 chromosomes of alfalfa, while another 11 *MsEXPs* were located on the unplaced genomic scaffolds. The tandem duplicated gene pairs are connected with red lines.

The genetic locations of the *MsEXPs* were mapped and labeled as *MsEXPA1–123*, *MsEXPB1–36*, *MsEXLA1–2*, and *MsEXLB1–18* in sequential chromosome order (Figure 2). In total, 157 out of 168 *MsEXPs* resided on 30 of the 32 alfalfa chromosomes, with the exception of chromosomes 6.1 and 6.4, and the remaining 11 *MsEXPs* were unassembled on the genomic scaffolds. The *MsEXPAs* were distributed between 30 chromosomes, with about 20.3% (25/123) residing on Chr 5 (Table S3). The *MsEXPBs* were located on three chromosomes, i.e., Chr 2, Chr 4, and Chr 7. For the *MsEXLs*, both *MsEXLAs* were found on Chr 4, and the *MsEXLBs* were found on Chr 5 or Chr 8. Although the physical location of the 11 unassembled *MsEXPAs* (113–123) was uncertain, the putative proteins belonged to EXPA-VI, suggesting that they may execute similar biological functions in alfalfa.

### 2.3. Gene Structure of the MsEXPs and Conserved Motifs of the Putative Proteins

**Figure 3:**
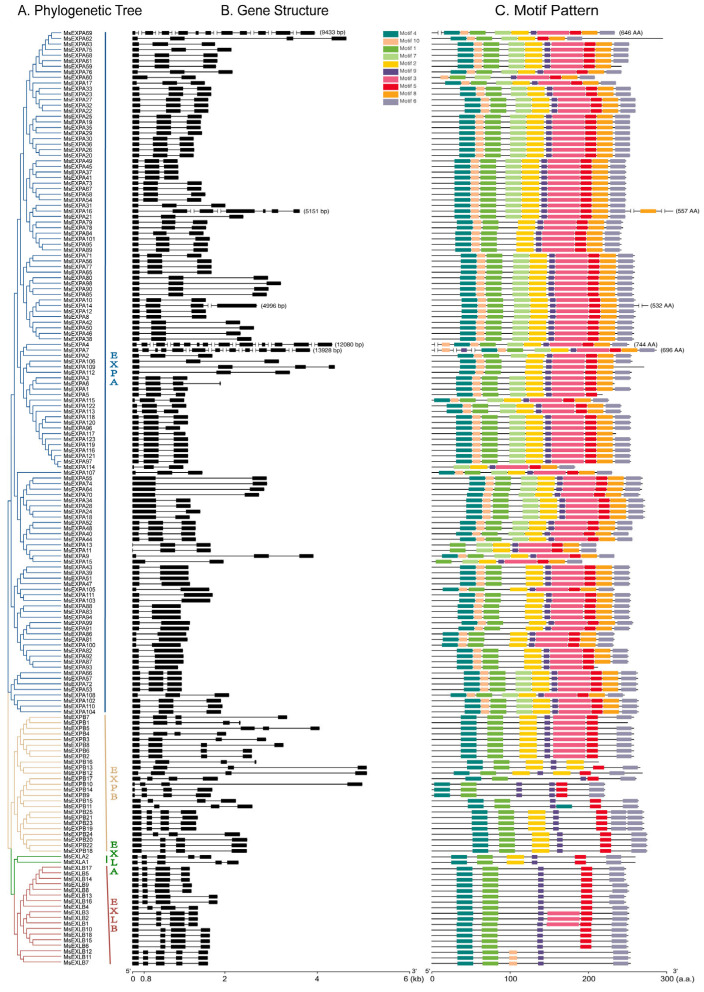
Phylogenetic tree, structural analysis, and motif distribution of the MsEXP family. (**A**) The phylogenetic tree was constructed based on the full-length sequences of MsEXPs and divided into four groups. Different subgroups are highlighted in different colors. (**B**) Exon–intron structures of *MsEXPs*. Introns and exons are represented by lines and boxes, respectively. (**C**) Analysis of MsEXP motifs is based on MEME tools. Different motifs are shown in colored boxes, as indicated in the legend.

According to the exon–intron composition analysis, the *MsEXP* members of the same subfamily share similar exon–intron structures (Figure 3A,B, Table S4). For example, 96% (24/25) of the *EXPBs* and 83.3% (15/18) of the *EXLBs* possess four exons. The majority (93.5%) of the *EXPA* members comprise two or three exons, with the exception of *MsEXPA4*, *MsEXPA7*, and *MsEXPA69*, which comprise 13, 12, and 11 exons, respectively (Figure 3A,B). 

According to the analysis of the conserved motifs from MEME (https://meme-suite.org/meme/, accessed on 22 February 2023), ten motifs (1–10) were found in the MsEXPs. Among them, Motifs 4 and 1 reside at the N-terminus, Motifs 6 and 5 reside at the C-terminus, and Motif 9 resides in the middle of most MsEXPs. Motifs 1, 3, and 6 feature the conserved domains (DPBB domain, RlpA-like domain, and cellulose-binding-like domain) of MsEXPs (Table S5). The members of the same subfamily share similar motifs arranged in identical order (Figure 3C). For example, 67.5% (83/123) of MsEXPA members share 10 motifs, while MsEXLBs possess 5–6 motifs. In the former, Motifs 7 and 8 are exclusive, while in the latter, Motifs 2, 7, and 8 are absent.

### 2.4. Gene Duplication and Synteny Analysis of MsEXPs

**Figure 4:**
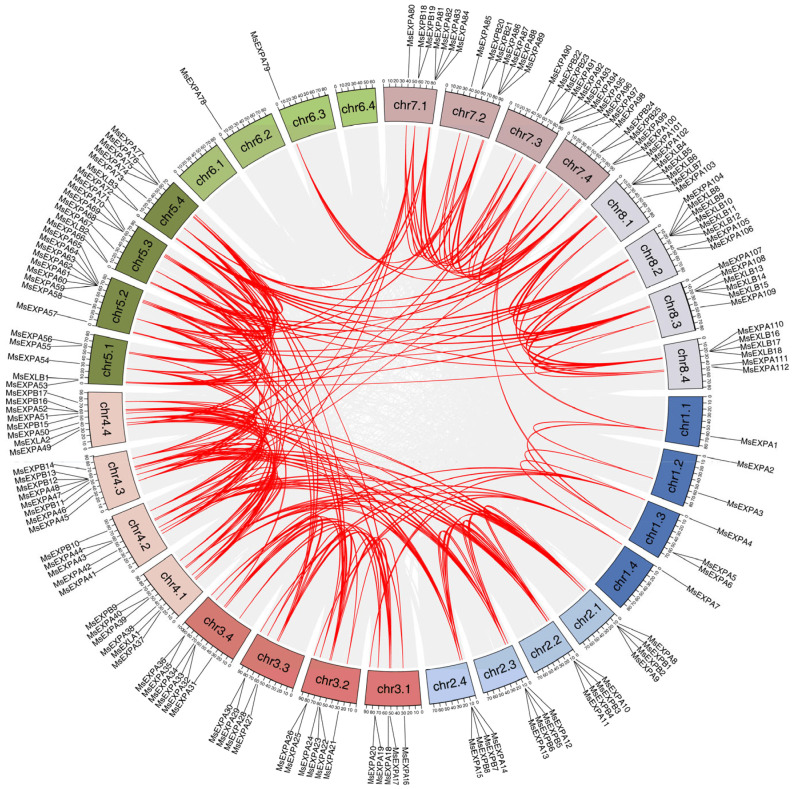
Collinearity analysis of *MsEXPs*. The identification syntenic relationship in *MsEXPs* is performed using TBtools software v1.108 (E-value ≤ 1 × 10^−5^). The gray lines show the syntenic regions in alfalfa genome. The genes pairs in segmental duplication events are marked with red lines.

According to the physical location of the 168 *MsEXPs*, 45 *MsEXPs* formed 22 tandem duplication events, including 21 pairs and one of three *MsEXP* paralogs (*MsEXPA81*, *MsEXPA82*, and *MsEXPA83*). Duplications were found on Chr2, Chr3, Chr7, and Chr8 and on scaffolds (Figure 2, Table S6). Notably, the homology between the eight tandemly duplicated genes on scaffolds was 93.9% (Figure S1), indicating that the two sets of tandem duplication genes may reside on the four homoeologous chromosomes. Moreover, a total of 250 segmental duplication events involving 121 *MsEXPs* were found, while the remaining 47 *MsEXPs* were singletons in the alfalfa genome (Figure 4, Table S7). Of these, approximately 60.4% (151/250) involved duplications of the four homoeologous chromosomes, such as *MsEXPB1* (10.8 Mb on Chr 2.1), *MsEXPB3* (8.9 Mb on Chr 2.2), *MsEXPB5* (10.3 Mb on Chr 2.3), and *MsEXPB7* (10.5 Mb on Chr 2.4). Thus, both tandem duplication and segmental duplication contribute to the expansion of the MsEXP family in alfalfa, and it is likely that the latter plays a dominant role. 

**Figure 5:**
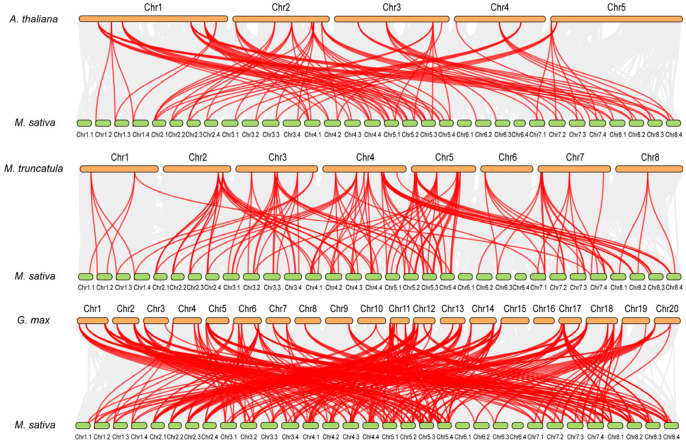
Synteny analysis of *Expansin* genes between alfalfa and three model plant species. The chromosome labels are located above or below the corresponding chromosome. Gray lines in the background indicate collinear blocks within alfalfa and the indicated plant, whereas the red lines highlight syntenic *Expansin* gene pairs.

To explore the evolutionary origin of *MsEXPs*, the genome collinearity among the three model plants, i.e., *Arabidopsis*, *M. truncatula*, soybean, and alfalfa, was compared [56]. Among the 168 *MsEXPs*, about 35.7% (60/168), 62.5% (105/168), and 66.7% (112/168) showed a collinear relationship with *Arabidopsis*, barrel clover, and soybean, respectively (Figure 5, Table S8). *MsEXPs* shared 97, 139, and 319 orthologs with *Arabidopsis* (Table S8-1), *M. truncatula* (Table S8-2), and soybean (Table S8-3), respectively, which indicated that the three legumes are close relatives. Interestingly, this was not the case for the MsEXPB subfamily, in which the proportion of orthologs shared with *MtEXPBs* (40%, 10/25) was lower than that shared with *AtEXPBs* (52%, 13/25) (Table S8-4). 

### 2.5. The Cis-Acting Elements Related to Abiotic Stresses Were Enriched in the Putative Promoters of MsEXPs

**Figure 6:**
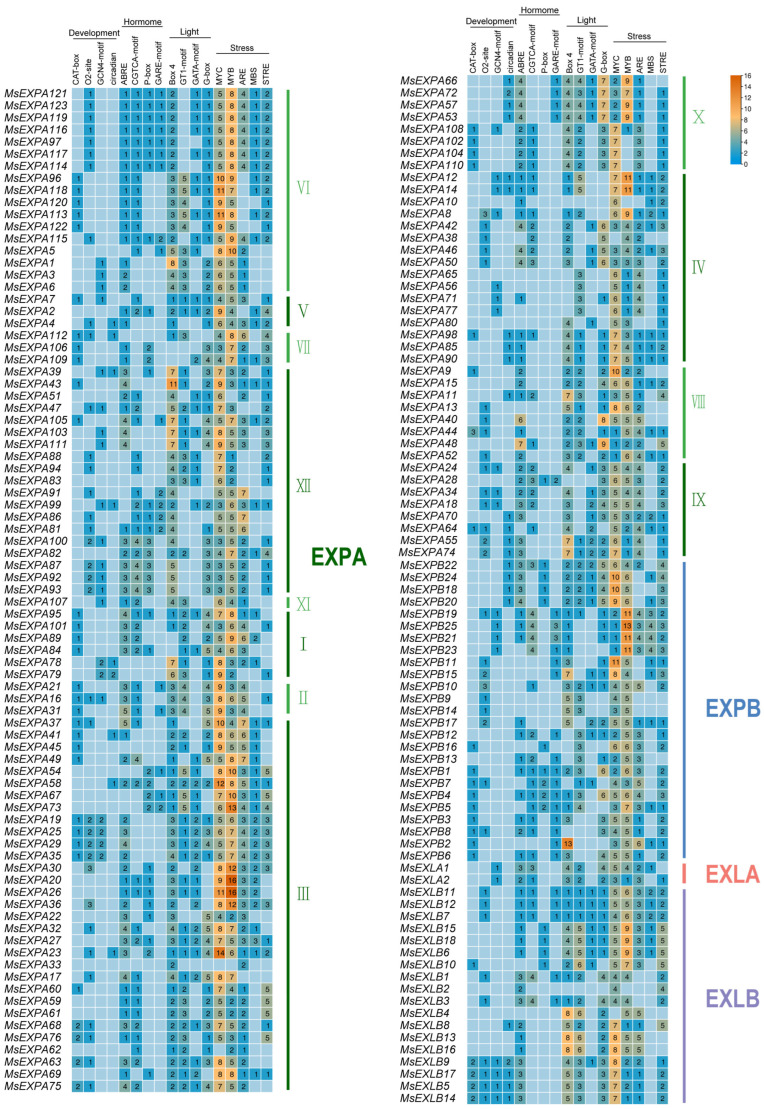
A heatmap showing the counts of *cis*-acting elements in the promoters of *MsEXPs*. The *MsEXPs* are grouped according to the phylogenetic results, with the green, blue, tangerine, and purple vertical lines referring to the EXPA, EXPB, EXLA, and EXLB phylogenetic groups, respectively. EXPAI–XII labels are alternately marked with dark green or green. The digit in the box represents the number of *cis*-acting elements (at the top).

To predict the potential function of the *MsEXPs*, we analyzed the *cis*-acting elements in the putative promoter region (2 kb upstream of the start codon) using PlantCARE (http://bioinformatics.psb.ugent.be/webtools/plantcare/html/, accessed on 24 February 2023). A total of 113 *cis*-acting elements were identified (Table S9-1), which were roughly divided into four major classes related to the following physiological processes: growth and development, plant hormones, light responsiveness, and abiotic stresses (Table S9-3). The top four to five elements of each class are shown in Figure 6 (Table S9-2). Among them, the stress-responsive elements accounted for 56.3% (2657/4719), while MYC and MYB accounted for 36% (956/2657) and 33.9% (900/2657), respectively. It is tempting to speculate that the MsEXP family may play a crucial role in the response of alfalfa to abiotic stresses. 

### 2.6. The Expression of MsEXPs under Drought and Salt Stress

**Figure 7:**
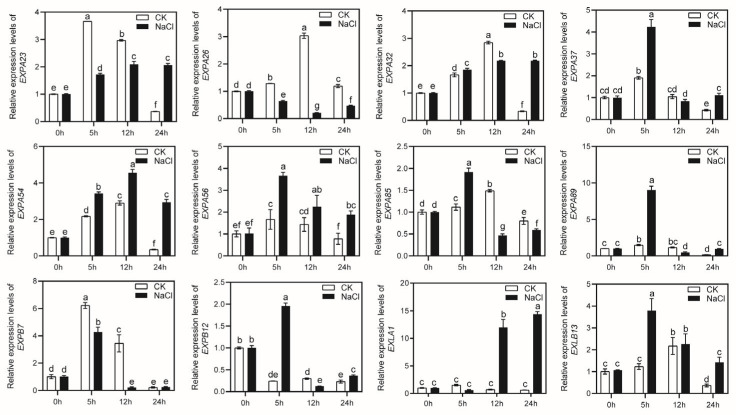
Expression levels of *MsEXPs* under salt stress. Four-week-old alfalfa seedlings were treated. “CK” represents 1/2 Hoagland solution. “NaCl” represents salt treatment using 1/2 Hoagland solution with 200 mM NaCl. The error bars indicate the standard errors of three biological replicates. Lowercase letters indicate significant differences at *p* < 0.05 according to ANOVA.

**Figure 8:**
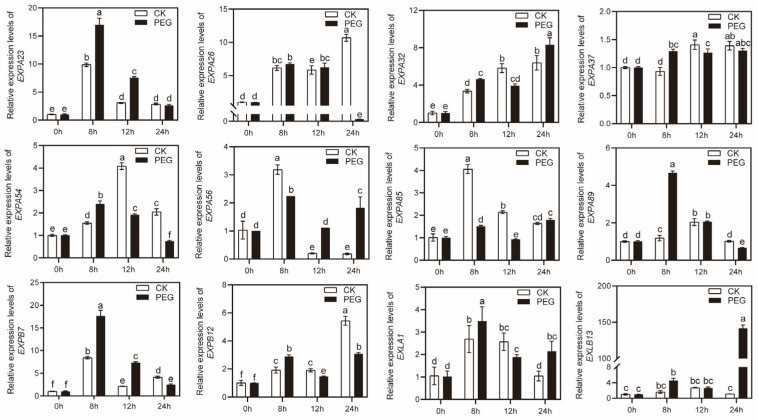
Expression levels of *MsEXPs* under drought stress. Four-week-old alfalfa seedlings were treated. “CK” represents 1/2 Hoagland solution. “PEG” represents drought treatment using 1/2 Hoagland solution with 15% PEG (polyethylene glycol). The error bars indicate the standard errors of three biological replicates. Lowercase letters indicate significant differences at *p* < 0.05 according to ANOVA.

In order to investigate the expression levels of *MsEXPs* under short-term drought and salt stress conditions, four-week-old alfalfa seedlings were treated with 200 mM NaCl or 15% PEG (polyethylene glycol) simulated drought at the time points of 0 h, 5 h/8 h, 12 h, and 24 h. Twelve *MsEXPs* were randomly selected, and their transcriptional levels were determined using qRT-PCR. Our results showed that about 83.3% of the 12 *MsEXPs* in the alfalfa seedlings were significantly upregulated when the salinity treatment was undertaken at different time points, while *MsEXPA26* was uniformly downregulated during the treatment periods (Figure 7). For the drought treatment, 15% PEG was used to induce the 12 *MsEXPs* at varying degrees (Figure 8). It is worth noting that during the 24 h PEG treatment, the transcriptional level of *MsEXLB13* was 127.7 times higher than that of the non-treated group. These findings indicated that the *MsEXPs* responded to both high-salinity and drought conditions. In addition, *MsEXLB13* may serve as a potential target for drought-related processes. Collectively, the transcriptional abundance of *MsEXPs* was altered in response to short-term drought and salt stress.

## 3. Discussion

Since *Expansin* genes (*EXPs*) were discovered in cucumber hypocotyls, an increasing number of studies have shown that they play key roles in the growth, development, and abiotic and biotic stress responses of a variety of plant species [11,48,57]. We focused on *Expansins* in forage legumes based on the genome data cultivar “XinjiangDaye” and identified the potential *MsEXPs* using comprehensive bioinformatics analysis; in seedlings, dozens of *MsEXPs* displayed significant alterations at the transcriptional level upon exposure to salt or drought treatment for a short-term period (<24 h). It is tempting to speculate that *MsEXPs* are implicated in forage adaptation to environmental cues. 

MsEXPs, together with MtEXPs and AtEXPs, are composed of the EXPA, EXPB, EXLA, and EXLB subfamilies. Of the 168 putative alfalfa *Expansins* or *Expansin-like* genes (*MsEXPs*), 123 were classified as α-Expansin (EXPA), 25 as β-Expansin (EXPB), 2 as Expansin-like A (EXLA), and 18 as Expansin-like B (EXLB). The proportions of the four MsEXP categories were similar to those of the model plants, i.e., *Arabidopsis*, barrel clover, and soybean. Generally, EXPA family members accounted for the majority (50–80%), while the EXLA family accounted for the least (Table S11). Alfalfa showed more MsEXPs than in wild and cultivated soybean (75), possibly due to the tetraploid nature of the former. MsEXPs contain the conservative domains double-psi beta-barrel domain-1 (DPBB_1) and Pollen-allerg_1. The former has a structure similar to the Family 45 glycoside hydrolases (GH45), but lacks the activity of the GH45 enzymes β-1 and 4-glucanase hydrolase [18,58]. The latter is a pollen allergen that can loosen the cell walls of the stigma and style to aid pollen tube penetration [59]. The DPBB_1 domain is likely to combine with the Pollen_allerg_1 component to complete cell-wall-loosening activity [60]. The expansion of the MsEXP family is likely to have resulted from both tandem and segmental duplication events in alfalfa. Previous studies have suggested that EXPAs and EXPBs evolved before the differentiation of vascular plants and mosses, while EXLAs and EXLBs appear to have emerged in the ancestors of angiosperms and gymnosperms [25]. In alfalfa, the four Expansin subfamilies are present. Relative to *MsEXPAs*, which account for 73.2% of *MsEXPs*, the members of the other three subfamilies appear to share a higher similarity in terms of both sequence homology and intron–exon structure, implying the functional redundancy of the *MsEXP* paralogs and the potential divergence of the paralogs among different subfamilies. MsEXPA members, like their orthologs in *Arabidopsis*, contain fewer introns [61], and the individual MsEXPA group shares a higher identity. In the gene structure of alfalfa *EXPs*, the numbers of introns and conserved motifs were nearly identical for the members of the same subfamily, implying a close evolutionary relationship. In contrast, the gene composition of members from different MsEXP subfamilies was distinct, and some conserved motifs were specific to certain subfamilies, implying the divergence of the biological functions of the four alfalfa EXP subfamilies. This suggests that *MsEXPs* have evolved multiple functions in alfalfa cultivation [15]. Our finding that 98.8% of MsEXPs are located on the cell wall suggests that MsEXPs are involved in material transport and resisting external environmental invasion [62,63]. 

In China, alfalfa has been planted in both arid and saline alkali areas, particularly in the northern part of the nation [64], and, therefore, it is vital to explore the genes implicated in salt or drought tolerance in alfalfa cultivation and production. The cell wall of plants is the first barrier to respond to and defend against external environmental stresses via alterations in its structure or composition, implying the potential of *Expansins* in stress response processes [65]. The key *cis*-regulatory elements in *MsEXP* promoters are related to light and abiotic stress responses, the majority of which are MYB, MYC, and other elements related to salt and drought induction, suggesting their possible role in salt and drought stresses. Our results are supportive of previous reports that MYC and MYB proteins play a transcriptional activation role in inducing gene expression under abiotic stress in plants [66,67]. The transcription of several candidate *Expansin* genes changed differently under NaCl or drought treatments. Most *MsEXPs* were upregulated at different degrees of high salinity. For PEG (15%)-mimicked drought treatment, the tested *MsEXPs* showed upregulated expression at different time points. This is consistent with previous findings that the overexpression of *AtEXLA18* improves the drought resistance of transgenic tobacco plants [57]. Therefore, it is tempting to speculate that some *EXPs* contribute to the induction of cell wall expansion within a short time period under salt and drought stress, which results in the improvement of water-use efficiency and a reduction in the internal water potential in cells, allowing plants to absorb extra water and mitigate stress damage. These findings suggest the potential implication of *Expansins* in alfalfa adaption to high salinity and drought. Future studies will focus on the response of *MsEXPs* to long-term (>24 h) stress treatment; further generation and investigation of loss-of-function mutations in transgenic plants would provide molecular evidence for the biological functions of *MsEXPs* in alfalfa in response to abiotic stresses. 

## 4. Materials and Methods

### 4.1. Identification of MsEXPs

The protein sequences and coding sequences (CDSs) of *A. thaliana* were obtained from TAIR (https://www.arabidopsis.org/, accessed on 8 January 2023). The genome and annotation information of alfalfa (Cultivar “XinjiangDaYe”) was downloaded from the figshare data repository (https://figshare.com/projects/whole_genome_sequencing_and_assembly_of_Medicago_sativa/66380, accessed on 16 September 2022), the *M. truncatula* from http://www.medicagogenome.org/, accessed on 16 September 2022, and the *G. max* from https://www.soybase.org/dlpages/, accessed on 16 September 2022. The 36 AtEXPs were used as query sequences to perform BlastP in TBtools software v1.108 (Chen, C., Guangzhou, China) [68] with 1 × 10^−5^ cutoff E-values against the alfalfa reference genome. The MsEXPs were examined using the Hidden Markov Model (HMM) in Pfam (http://pfam-legacy.xfam.org/, accessed on 16 January 2023 [69]), and an E-value of 1.0 was set as the threshold to ensure that these MsEXPs contained the conserved domains (DPBB_1 PF03330 and Pollen_allerg_1 PF01357) and remove proteins without characteristic domains. The protein sequence length, molecular weight (MW), and isoelectric points (pIs) were obtained from ExPaSy (https://www.expasy.org/, accessed on 19 February 2023 [70]). Subcellular localization prediction was performed using Plant-PLoc (http://www.csbio.sjtu.edu.cn/bioinf/plant/, accessed on 19 February 2023 [71]). 

### 4.2. Phylogenetic Analysis and Comparison of MsEXPs

Using the ClustalW program in MEGA 7.0 software (Tamura, K., Tokyo, Japan) [72], the full-length amino acid sequences were aligned, and the phylogenetic tree was built with EXP protein sequences from alfalfa, *M. truncatula*, and *Arabidopsis* using the neighbor-joining (NJ) method (bootstrap values for 1000 replicates) and visualized using Evolview v2 (https://www.evolgenius.info/evolview-v2/, accessed on 10 January 2024). 

### 4.3. Chromosomal Location and Structural Characterization Analysis of MsEXPs

The information on the location of the *MsEXPs* was obtained from the alfalfa genome database. All *MsEXPs* were mapped to the chromosomes using MG2C (http://mg2c.iask.in/, accessed on 27 January 2024). Conserved motifs in the MsEXPs were analyzed using the MEME online program (https://meme-suite.org/meme/, accessed on 22 February 2023), while the optimum motif width was set to ≥6 bp and ≤50 bp, and the maximum number of motifs was set to 10; all other parameters were used as default values. The structure (exon–intron) distributions of the *MsEXPs* were obtained from the genome of alfalfa using GFF annotation files. The results were visualized using TBtools software v1.108 (Chen, C., Guangzhou, China) [68]. 

### 4.4. Analysis of Cis-Acting Elements of MsEXPs

The assumed promoter region of the *MsEXP* genes (2 kb upstream of the coding region) was obtained from the alfalfa genome database. *Cis*-acting components were predicted using the PlantCARE online software (http://bioinformatics.psb.ugent.be/webtools/plantcare/html/, accessed on 24 February 2023), and the predicted results were visualized using TBtools software v1.108 (Chen, C., Guangzhou, China) [68].

### 4.5. Gene Duplication Pattern and Collinearity Analysis of MsEXPs

The identification of the gene duplication events and syntenic relationships of *MsEXPs* was performed using the one-step MCScanX–Super fast program in the TBtools software v1.108 (Chen, C., Guangzhou, China) (E-value ≤ 1 × 10^−5^) [68]. The tandem duplicated genes within 200 kb were defined as adjacent homologous genes as long as the following two criteria were met: (i) the length of the aligned sequence covered >75% of the longer gene, and (ii) the similarity of the aligned regions was >75% with no more than two intervening genes [16,73]. The collinear regions between *MsEXPs* and *EXPs* from alfalfa, *Arabidopsis*, *M. truncatula*, and soybean were visualized using the Dual Synteny Plotter and Advanced Circos function in the TBtools software v1.108 (Chen, C., Guangzhou, China). 

### 4.6. Plant Material Growth Conditions and Stress Treatment

Alfalfa (Cultivar “Zhongmu No. 1”) was used in this study. Mature seeds were germinated on moist filter paper with ultrapure water in Petri dishes and grown in a growth chamber (GXZ-500, Ningbo Jiangnan Instrument Factory, Ningbo, China) at 24 °C (day)/20 °C (night) under a 16 h light (1200–1250 µmol m^−2^s^−1^)/8 h dark photoperiod with a relative humidity of 70 to 80% for 7 days. After germination, the alfalfa seedlings were transferred to a plastic cuboid container (25 cm × 20 cm × 7.5 cm) with 1/2 Hoagland nutrient solution under controlled conditions, and the nutrient solution was replaced once a week. In order to assay the salt stress tolerance, the four-week-old alfalfa seedlings were treated with and without 200 mM NaCl for 0, 5, 12, and 24 h. For the drought stress tolerance expression assay, the four-week-old alfalfa seedings were used as the planting material for the stress treatment with or without 15% PEG-6000 for 0, 8, 12, and 24 h. The leaf tissues of the alfalfa seedlings were collected, immediately placed in liquid nitrogen, and stored at −80 °C until determination. 

### 4.7. RNA Extraction and Real-Time Quantitative PCR (qRT-PCR)

The total RNA was extracted with a Plant total RNA extract kit (Promega, Madison, WI, USA) according to the manufacturer’s instructions. RNA concentration and purity were measured using the NanoPhotometer^®^ NP80 (Implen, Munich, Germany), and the quality detected using 1% agarose gel electrophoresis. The RNA concentrations ranged from 200 to 1000 ng/mL, and the OD260/OD280 ratios ranged from 1.8 to 2.2. The cDNA was synthesized from 1 μg total RNA using the HiScript III All-in-one RT SuperMix Perfect for qPCR (Vazyme, Nanjing, China), and cDNA was then diluted 10 times and stored at −20 °C before use. The expression patterns of *MsEXPs* in alfalfa seedlings treated with PEG or NaCl were examined (primers listed in Table S10) using the CFX96 Touch^TM^ RT-PCR system (BioRad, Los Angeles, CA, USA) with Taq Pro Universal SYBR qPCR Master Mix (Vazyme, Nanjing, China). The relative expression levels were calculated using the 2^−∆∆CT^ method [74]. 

### 4.8. Statistical Analysis

Statistical analyses were performed using SPSS 22 software (IBM Inc., Chicago, IL, USA). According to Duncan’s multiple range test, the differences were evaluated using ANOVA with *p* < 0.05. GraphPad Prism 9.0.0 software (GraphPad Software, Boston, FL, USA) was used to plot. Adobe Illustrator CC 2018 (ADOBE, San Jose, CA, USA)was used for graphic editing. 

## 5. Conclusions

In this study, we conducted a comprehensive genome-wide analysis of the Expansin family in alfalfa, decoding 168 *MsEXP*s. Phylogenetically, the MsEXPs were split into four subfamilies, EXPA, EXPB, EXLA, and EXLB, with the former two subfamilies representing the majority (88.1%). As expected, the members of each subfamily shared similar gene and motif compositions, implying identical functions. In the putative *MsEXP* promoters, 56.3% of the *cis*-elements were related to abiotic stress. In support of this, the transcriptional levels of *MsEXPs*, including *MsEXPA56/89/26*, *MsEXLB13*, and *MsEXLA1*, displayed a significant alteration in alfalfa seedlings treated by salt or drought compared with the non-treatment control. In summary, this study provides valuable insights into the evolution of *Expansin* genes in alfalfa and provides more information for exploring alterations in *MsEXP* transcripts under long-term stress that can be applied to forage breeding.

## Data Availability

Data are contained within the article and Supplementary Materials.

## Supplementary Materials

The following supporting information can be downloaded at: https://www.mdpi.com/article/10.3390/ijms25094700/s1.

## References

[1] Cosgrove D.J. (2005). Growth of the plant cell wall. Nat. Rev. Mol. Cell Biol..

[2] Zhang B., Gao Y., Zhang L., Zhou Y. (2021). The plant cell wall: Biosynthesis, construction, and functions. J. Integr. Plant Biol..

[3] Zhu J.K. (2016). Abiotic stress signaling and responses in plants. Cell.

[4] Chebli Y., Bidhendi A.J., Kapoor K., Geitmann A. (2021). Cytoskeletal regulation of primary plant cell wall assembly. Curr. Biol..

[5] Carpita N.C., Gibeaut D.M. (1993). Structural models of primary cell walls in flowering plants: Consistency of molecular structure with the physical properties of the walls during growth. Plant J..

[6] Vorwerk S., Somerville S., Somerville C. (2004). The role of plant cell wall polysaccharide composition in disease resistance. Trends Plant Sci..

[7] Wolf S., Mravec J., Greiner S., Mouille G., Höfte H. (2012). Plant cell wall homeostasis is mediated by brassinosteroid feedback signaling. Curr. Biol..

[8] Du F., Jiao Y. (2020). Mechanical control of plant morphogenesis: Concepts and progress. Curr. Opin. Plant Biol..

[9] Sun W., Yu H., Liu M., Ma Z., Chen H. (2021). Evolutionary research on the expansin protein family during the plant transition to land provides new insights into the development of Tartary buckwheat fruit. BMC Genom..

[10] Cosgrove D.J. (1989). Characterization of long-term extension of isolated cell walls from growing cucumber hypocotyls. Planta.

[11] McCann M.C., Wells B., Roberts K. (1992). Complexity in the spatial localization and length distribution of plant cell-wall matrix polysaccharides. J. Microsc..

[12] McQueen-Mason S., Durachko D.M., Cosgrove D.J. (1992). Two endogenous proteins that induce cell wall extension in plants. Plant Cell.

[13] Kende H., Bradford K., Brummell D., Cho H.T., Cosgrove D., Fleming A., Gehring C., Lee Y., McQueen-Mason S., Rose J. (2004). Nomenclature for members of the expansin superfamily of genes and proteins. Plant Mol. Biol..

[14] Cosgrove D.J., Bedinger P., Durachko D.M. (1997). Group I allergens of grass pollen as cell wall-loosening agents. Proc. Natl. Acad. Sci. USA.

[15] Han Z., Liu Y., Deng X., Liu D., Liu Y., Hu Y., Yan Y. (2019). Genome-wide identification and expression analysis of expansin gene family in common wheat (*Triticum aestivum* L.). BMC Genom..

[16] Li K., Ma B., Shen J., Zhao S., Ma X., Wang Z., Fan Y., Tang Q., Wei D. (2021). The evolution of the expansin gene family in *Brassica species*. Plant Physiol. Bioch..

[17] Lee Y., Choi D., Kende H. (2001). Expansins: Ever-expanding numbers and functions. Curr. Opin. Plant Biol..

[18] Yennawar N.H., Li L.C., Dudzinski D.M., Tabuchi A., Cosgrove D.J. (2006). Crystal structure and activities of *EXPB1* (*Zea m 1*), a beta-expansin and group-1 pollen allergen from maize. Proc. Natl. Acad. Sci. USA.

[19] Boron A.K., Van Loock B., Suslov D., Markakis M.N., Verbelen J.P., Vissenberg K. (2015). Over-expression of *AtEXLA2* alters etiolated arabidopsis hypocotyl growth. Ann. Bot..

[20] Guimaraes L.A., Mota A.P.Z., Araujo A.C.G., de Alencar Figueiredo L.F., Pereira B.M., de Passos Saraiva M.A., Silva R.B., Danchin E.G.J., Guimaraes P.M., Brasileiro A.C.M. (2017). Genome-wide analysis of expansin superfamily in wild *Arachis discloses* a stress-responsive expansin-like B gene. Plant Mol. Biol..

[21] Schipper O., Schaefer D., Reski R., Flemin A. (2002). Expansins in the bryophyte Physcomitrella patens. Plant Mol. Biol..

[22] Carey R.E., Cosgrove D.J. (2007). Portrait of the expansin superfamily in Physcomitrella patens: Comparisons with angiosperm expansins. Ann. Bot..

[23] Carey R.E., Hepler N.K., Cosgrove D.J. (2013). Selaginella moellendorffii has a reduced and highly conserved expansin superfamily with genes more closely related to angiosperms than to bryophytes. BMC Plant Biol..

[24] Guo F., Guo J., El-Kassaby Y.A., Wang G. (2023). Genome-wide identification of expansin gene family and their response under hormone exposure in *Ginkgo biloba* L.. Int. J. Mol. Sci..

[25] Sampedro J., Carey R.E., Cosgrove D.J. (2006). Genome histories clarify evolution of the expansin superfamily: New insights from the poplar genome and pine ESTs. J. Plant Res..

[26] Tan J., Wang M., Shi Z., Miao X. (2018). *OsEXPA10* mediates the balance between growth and resistance to biotic stress in rice. Plant Cell Rep..

[27] Santiago T.R., Pereira V.M., de Souza W.R., Steindorff A.S., Cunha B., Gaspar M., Fávaro L.C.L., Formighieri E.F., Kobayashi A.K., Molinari H.B. (2018). Genome-wide identification, characterization and expression profile analysis of expansins gene family in sugarcane (*Saccharum* spp.). PLoS ONE.

[28] Feng X., Li C., He F., Xu Y., Li L., Wang X., Chen Q., Li F. (2022). Genome-wide identification of expansin genes in wild soybean (*Glycine soja*) and functional characterization of Expansin B1 (*GsEXPB1*) in soybean hair root. Int. J. Mol. Sci..

[29] Zhu Y., Wu N., Song W., Yin G., Qin Y., Yan Y., Hu Y. (2014). Soybean (*Glycine max*) expansin gene superfamily origins: Segmental and tandem duplication events followed by divergent selection among subfamilies. BMC Plant Biol..

[30] Magadum S., Banerjee U., Murugan P., Gangapur D., Ravikesavan R. (2013). Gene duplication as a major force in evolution. J. Genet..

[31] Choi D., Lee Y., Cho H.T., Kende H. (2003). Regulation of expansin gene expression affects growth and development in transgenic rice plants. Plant Cell.

[32] Cho H.T., Cosgrove D.J. (2002). Regulation of root hair initiation and expansin gene expression in *Arabidopsis*. Plant Cell.

[33] Lee D.K., Ahn J.H., Song S.K., Choi Y.D., Lee J.S. (2003). Expression of an expansin gene is correlated with root elongation in soybean. Plant Physiol..

[34] Pien S., Wyrzykowska J., McQueen-Mason S., Smart C., Fleming A. (2001). Local expression of expansin induces the entire process of leaf development and modifies leaf shape. Proc. Natl. Acad. Sci. USA.

[35] Yan A., Wu M., Yan L., Hu R., Ali I., Gan Y. (2014). *AtEXP2* is involved in seed germination and abiotic stress response in Arabidopsis. PLoS ONE.

[36] Pezzotti M., Feron R., Mariani C. (2002). Pollination modulates expression of the PPAL gene, a pistil-specific beta-expansin. Plant Mol. Biol..

[37] Li X., Zhao J., Walk T.C., Liao H. (2014). Characterization of soybean β-expansin genes and their expression responses to symbiosis, nutrient deficiency, and hormone treatment. Appl. Microbiol. Biotechnol..

[38] Li X., Zhao J., Tan Z., Zeng R., Liao H. (2015). *GmEXPB2*, a cell wall β-Expansin, affects soybean nodulation through modifying root architecture and promoting nodule formation and development. Plant Physiol..

[39] Han Y., Li A., Li F., Zhao M., Wang W. (2012). Characterization of a wheat (*Triticum aestivum* L.) expansin gene, *TaEXPB23*, involved in the abiotic stress response and phytohormone regulation. Plant Physiol. Bioch..

[40] Muthusamy M., Kim J.Y., Yoon E.K., Kim J.A., Lee S.I. (2020). *BrEXLB1*, a *Brassica rapa* Expansin-Like B1 Gene is associated with root development, drought stress response, and seed germination. Genes.

[41] Dai F., Zhang C., Jiang X., Kang M., Yin X., Lü P., Zhang X., Zheng Y., Gao J. (2012). *RhNAC2* and *RhEXPA4* are involved in the regulation of dehydration tolerance during the expansion of rose petals. Plant Physiol..

[42] Marowa P., Ding A., Xu Z., Kong Y. (2020). Overexpression of *NtEXPA11* modulates plant growth and development and enhances stress tolerance in tobacco. Plant Physiol. Bioch..

[43] Han Y., Chen Y., Yin S., Zhang M., Wang W. (2015). Over-expression of *TaEXPB23*, a wheat expansin gene, improves oxidative stress tolerance in transgenic tobacco plants. J. Plant Physiol..

[44] Li F., Xing S., Guo Q., Zhao M., Zhang J., Gao Q., Wang G., Wang W. (2011). Drought tolerance through over-expression of the expansin gene *TaEXPB23* in transgenic tobacco. J. Plant Physiol..

[45] Harb A., Krishnan A., Ambavaram M.M., Pereira A. (2010). Molecular and physiological analysis of drought stress in *Arabidopsis* reveals early responses leading to acclimation in plant growth. Plant Physiol..

[46] Wolf S. (2017). Plant cell wall signalling and receptor-like kinases. Biochem. J..

[47] Bashline L., Lei L., Li S., Gu Y. (2014). Cell wall, cytoskeleton, and cell expansion in higher plants. Mol. Plant.

[48] Yang J., Zhang G., An J., Li Q., Chen Y., Zhao X., Wu J., Wang Y., Hao Q., Wang W. (2020). Expansin gene *TaEXPA2* positively regulates drought tolerance in transgenic wheat (*Triticum aestivum* L.). Plant Sci..

[49] He X., Zeng J., Cao F., Ahmed I.M., Zhang G., Vincze E., Wu F. (2015). *HvEXPB7*, a novel β-expansin gene revealed by the root hair transcriptome of Tibetan wild barley, improves root hair growth under drought stress. J. Exp. Bot..

[50] Sun W., Yao M., Wang Z., Chen Y., Zhan J., Yan J., Jiang S., Jian S., Chen H., Bu T. (2022). Involvement of auxin-mediated *CqEXPA50* contributes to salt tolerance in Quinoa (*Chenopodium quinoa*) by interaction with auxin pathway genes. Int. J. Mol. Sci..

[51] Geilfus C.M., Ober D., Eichacker L.A., Mühling K.H., Zörb C. (2015). Down-regulation of *ZmEXPB6* (Zea mays β-expansin 6) protein is correlated with salt-mediated growth reduction in the leaves of *Z. mays* L.. J. Biol. Chem..

[52] Peng L.N., Xu Y.Q., Wang X., Feng X., Zhao Q.Q., Feng S.S., Zhao Z.Y., Hu B.Z., Li F.L. (2019). Overexpression of paralogues of the wheat expansin gene *TaEXPA8* improves low-temperature tolerance in *Arabidopsis*. Plant Biol..

[53] Feng X., Xu Y., Peng L., Yu X., Zhao Q., Feng S., Zhao Z., Li F., Hu B. (2019). *TaEXPB7-B*, a β-expansin gene involved in low-temperature stress and abscisic acid responses, promotes growth and cold resistance in *Arabidopsis thaliana*. J. Plant Physiol..

[54] Chen H., Zeng Y., Yang Y., Huang L., Tang B., Zhang H., Hao F., Liu W., Li Y., Liu Y. (2020). Allele-aware chromosome-level genome assembly and efficient transgene-free genome editing for the autotetraploid cultivated alfalfa. Nat. Commun..

[55] Sampedro J., Cosgrove D.J. (2005). The expansin superfamily. Genome Biol..

[56] Wang Y., Li J., Paterson A.H. (2013). MCScanX-transposed: Detecting transposed gene duplications based on multiple colinearity scans. Bioinformatics.

[57] Abbasi A., Malekpour M., Sobhanverdi S. (2021). The *Arabidopsis* expansin gene (*AtEXPA18*) is capable to ameliorate drought stress tolerance in transgenic tobacco plants. Mol. Biol. Rep..

[58] Castillo R.M., Mizuguchi K., Dhanaraj V., Albert A., Blundell T.L., Murzin A.G. (1999). A six-stranded double-psi beta barrel is shared by several protein superfamilies. Structure.

[59] Jiang S.Y., Jasmin P.X., Ting Y.Y., Ramachandran S. (2005). Genome-wide identification and molecular characterization of Ole_e_I, Allerg_1 and Allerg_2 domain-containing pollen-allergen-like genes in *Oryza sativa*. DNA Res..

[60] Cosgrove D.J. (2015). Plant expansins: Diversity and interactions with plant cell walls. Curr. Opin. Plant Biol..

[61] Liu W., Lyu T., Xu L., Hu Z., Xiong X., Liu T., Cao J. (2020). Complex molecular evolution and expression of Expansin gene families in three basic diploid species of *Brassica*. Int. J. Mol. Sci..

[62] Wu W., Zhu S., Chen Q., Lin Y., Tian J., Liang C. (2019). Cell wall proteins play critical roles in plant adaptation to phosphorus deficiency. Int. J. Mol. Sci..

[63] Narváez-Barragán D.A., Tovar-Herrera O.E., Segovia L., Serrano M., Martinez-Anaya C. (2020). Expansin-related proteins: Biology, microbe-plant interactions and associated plant-defense responses. Microbiology.

[64] Ling L., An Y., Wang D., Tang L., Du B., Shu Y., Bai Y., Guo C. (2022). Proteomic analysis reveals responsive mechanisms for saline-alkali stress in alfalfa. Plant Physiol. Bioch..

[65] Bacete L., Mélida H., Miedes E., Molina A. (2018). Plant cell wall-mediated immunity: Cell wall changes trigger disease resistance responses. Plant J..

[66] Abe H., Urao T., Ito T., Seki M., Shinozaki K., Yamaguchi-Shinozaki K. (2003). *Arabidopsis AtMYC2* (bHLH) and *AtMYB2* (MYB) function as transcriptional activators in abscisic acid signaling. Plant Cell.

[67] Wang X., Niu Y., Zheng Y. (2021). Multiple functions of MYB transcription factors in abiotic stress responses. Int. J. Mol. Sci..

[68] Chen C., Chen H., Zhang Y., Thomas H.R., Frank M.H., He Y., Xia R. (2020). TBtools: An integrative toolkit developed for interactive analyses of big biological data. Mol. Plant.

[69] Mistry J., Chuguransky S., Williams L., Qureshi M., Salazar G.A., Sonnhammer E.L.L., Tosatto S.C.E., Paladin L., Raj S., Richardson L.J. (2021). Pfam: The protein families database in 2021. Nucleic Acids Res..

[70] Duvaud S., Gabella C., Lisacek F., Stockinger H., Ioannidis V., Durinx C. (2021). Expasy, the Swiss Bioinformatics Resource Portal, as designed by its users. Nucleic Acids Res..

[71] Chou K.C., Shen H.B. (2008). Cell-PLoc: A package of Web servers for predicting subcellular localization of proteins in various organisms. Nat. Protoc..

[72] Kumar S., Stecher G., Tamura K. (2016). MEGA7: Molecular Evolutionary Genetics Analysis Version 7.0 for Bigger Datasets. Mol. Biol. Evol..

[73] Holub E.B. (2001). The arms race is ancient history in *Arabidopsis*, the wildflower. Nat. Rev. Genet..

[74] Livak K.J., Schmittgen T.D. (2001). Analysis of relative gene expression data using real-time quantitative PCR and the 2(-Delta Delta C(T)) Method. Methods.

