# Supplementary material for: Genome-Wide Identification, Phylogenetic and Expression Analysis of Expansin Gene Family in Medicago sativa L
Source: Int J Mol Sci. 2024 Apr 25;25(9):4700. doi: 10.3390/ijms25094700 (PMC11083626; doi:10.3390/ijms25094700)
Supplement: Supplementary file 1 [file ijms-25-04700-s001.zip › Supplementary Materials.pdf]

Genome-Wide Identification, Phylogenetic and Expression Analysis of Expansin Gene Family in *Medicago sativa* L.

Yajing Li [1, †], Yangyang Zhang [1, 2, †], Jing Cui [1], Xue Wang [1], Mingna Li [1], Lili Zhang [1], Zhen Wang [3] and Junmei Kang [1,*]

1 Institute of Animal Science, Chinese Academy of Agricultural Sciences, Beijing 100193, China;

ly_yajing@yeah.net (Y.L.); yang20170115@163.com (Y.Z.); cuijing0417@yeah.net (J.C.); wangxue01@caas.cn (X.W.); limingna@caas.cn (M.L.); lili_chang721@yeah.net (L.Z.);

2 College of Grassland Agriculture, Northwest A&F University, Yangling, 712100, Shaanxi, China;

3 Department of Agronomy and Horticulture, University of Nebraska-Lincoln, Lincoln, NE 68583, USA;

zwunl3@gmail.com (Z.W.);

* Correspondence: kangjunmei@caas.cn (J.K.);

† These authors contributed equally to this work.

```
MsEXPA113   ............MNMVTKELRVESAVWQQAHATFYGGSDASGTMGGACGYGNLYTDGYGIKSAALSTALFNDGKSCGGCYQIVCDARQVP    78
MsEXPA114   ................................................................MVEVMPQEQ.......WIVCDARQVP    19
MsEXPA118   MEKFIICVLLLLMNMVTKELRVESAVWQQAHATFYGGSDASGTMGGACGYGNLYTDGYGIKSAALSTALFNDGKSCGGCYQIVCDARQVP    90
MsEXPA119   MEKIIICVLILLINLLTKELRVESVVWQQAHATFYGGSDASGTMGGACGYGNLYTDGYGIKSAALSTALFNDGKSCGGCYQIVCDARQVP    90
MsEXPA120   MEKFIICVLLLLMNMVTKELRVESAVWQQAHATFYGGSDASGTMGGACGYGNLYTDGYGIKSAALSTALFNDGKSCGGCYQIVCDARQVP    90
MsEXPA121   MEKIIICVLILLINLLTKELRVESVVWQQAHATFYGGSDASGTMGGACGYGNLYTDGYGIKSAALSTALFNDGKSCGGCYQIVCDARQVP    90
MsEXPA122   ............MNMVTKELRVESAVWQQAHATFYGGSDASGTMGGACGYGNLYTDGYGIKSAALSTALFNDGKSCGGCYQIVCDARQVP    78
MsEXPA123   MEKIIICVLILLINLLTKELRVESVVWQQAHATFYGGSDASGTMGGACGYGNLYTDGYGIKSAALSTALFNDGKSCGGCYQIVCDARQVP    90
Consensus                                                                                    ivcdarqvp

MsEXPA113   QWCLRGTSITITATNFCPPNFALPNDNGGWCNPPRPHFDMSQPAFQTIAKYRAGIVPILYRRVGCKRSGNIRFTINGRDYFELVLISNVG   168
MsEXPA114   QWCLRGTSITITATNFCPPNFALPNDNGGWCNPPRPHFDMSQPAFQTIAKYRAGIVPILYRRVGCKRSGNIRFTINGRDYFELVLISNVG   109
MsEXPA118   QWCLRGTSITITATNFCPPNFALPNDNGGWCNPPRPHFDMSQPAFQTIAKYRAGIVPILYRRVGCKRSGNIRFTINGRDYFELVLISNVG   180
MsEXPA119   QWCLRGTSITITATNFCPPNFALPNDNGGWCNPPRPHFDMSQPAFQTIAKYRAGIVPILYRRVGCKRSGNIRFTINGRDYFELVLISNVG   180
MsEXPA120   QWCLRGTSITITATNFCPPNFALPNDNGGWCNPPRPHFDMSQPAFQTIAKYRAGIVPILYRRVGCKRSGNIRFTINGRDYFELVLISNVG   180
MsEXPA121   QWCLRGTSITITATNFCPPNFALPNDNGGWCNPPRPHFDMSQPAFQTIAKYRAGIVPILYRRVGCKRSGNIRFTINGRDYFELVLISNVG   180
MsEXPA122   QWCLRGTSITITATNFCPPNFALPNDNGGWCNPPRPHFDMSQPAFQTIAKYRAGIVPILYRRVGCKRSGNIRFTINGRDYFELVLISNVG   168
MsEXPA123   QWCLRGTSITITATNFCPPNFALPNDNGGWCNPPRPHFDMSQPAFQTIAKYRAGIVPILYRRVGCKRSGNIRFTINGRDYFELVLISNVG   180
Consensus   qwclrgtsititatnfcppnfalpndnggwcnpprphfdmsqpafqtiakyragivpilyrrvgckrsgnirftingrdyfelvlisnvg

MsEXPA113   GGGEISKVWIKGSKKNKWEPMSMNWGANWQSLSYLNGQSLSFRIQLKNGKTRTAINVAPSNWRFGQSYKSNVQ                     241
MsEXPA114   GGGEISKVWIKGSKKNKWEPMSMNWGANWQSLSYLNGQSLSFRIQLKNGKTRTAINVAPSNWRFGQSYKSNVQ                     182
MsEXPA118   GGGEISKVWIKGSKKNKWEPMSMNWGANWQSLSYLNGQSLSFRIQLKNGKTCTAINVAPSNWRFGQSYKSNVQ                     253
MsEXPA119   GGGEISKVWIKGSKKNKWEPMSMNWGANWQSLSYLNGQSLSFRIQLKNGKTRTAINVAPSNWRFGQSYKSNVQ                     253
MsEXPA120   GGGEISKVWIKGSKKNKWEPMSMNWGANWQSLSYLNGQSLSFRIQLKNGKTCTAINVAPSNWRFGQSYKSNVQ                     253
MsEXPA121   GGGEISKVWIKGSKKNKWEPMSMNWGANWQSLSYLNGQSLSFRIQLKNGKTRTAINVAPSNWRFGQSYKSNVQ                     253
MsEXPA122   GGGEISKVWIKGSKKNKWEPMSMNWGANWQSLSYLNGQSLSFRIQLKNGKTCTAINVAPSNWRFGQSYKSNVQ                     241
MsEXPA123   GGGEISKVWIKGSKKNKWEPMSMNWGANWQSLSYLNGQSLSFRIQLKNGKTRTAINVAPSNWRFGQSYKSNVQ                     253
Consensus   gggeiskvwikgskknkwepmsmnwganwqslsylngqslsfriqlkngkt tainvapsnwrfgqsyksnvq
```

Figure S1. Homology comparison among eight tandem duplication genes on the scaffold.
