# Supplementary figures and images for: Genome-Wide Identification, Phylogenetic and Expression Analysis of Expansin Gene Family in Medicago sativa L
Source: Int J Mol Sci. 2024 Apr 25;25(9):4700. doi: 10.3390/ijms25094700 (PMC11083626; doi:10.3390/ijms25094700)

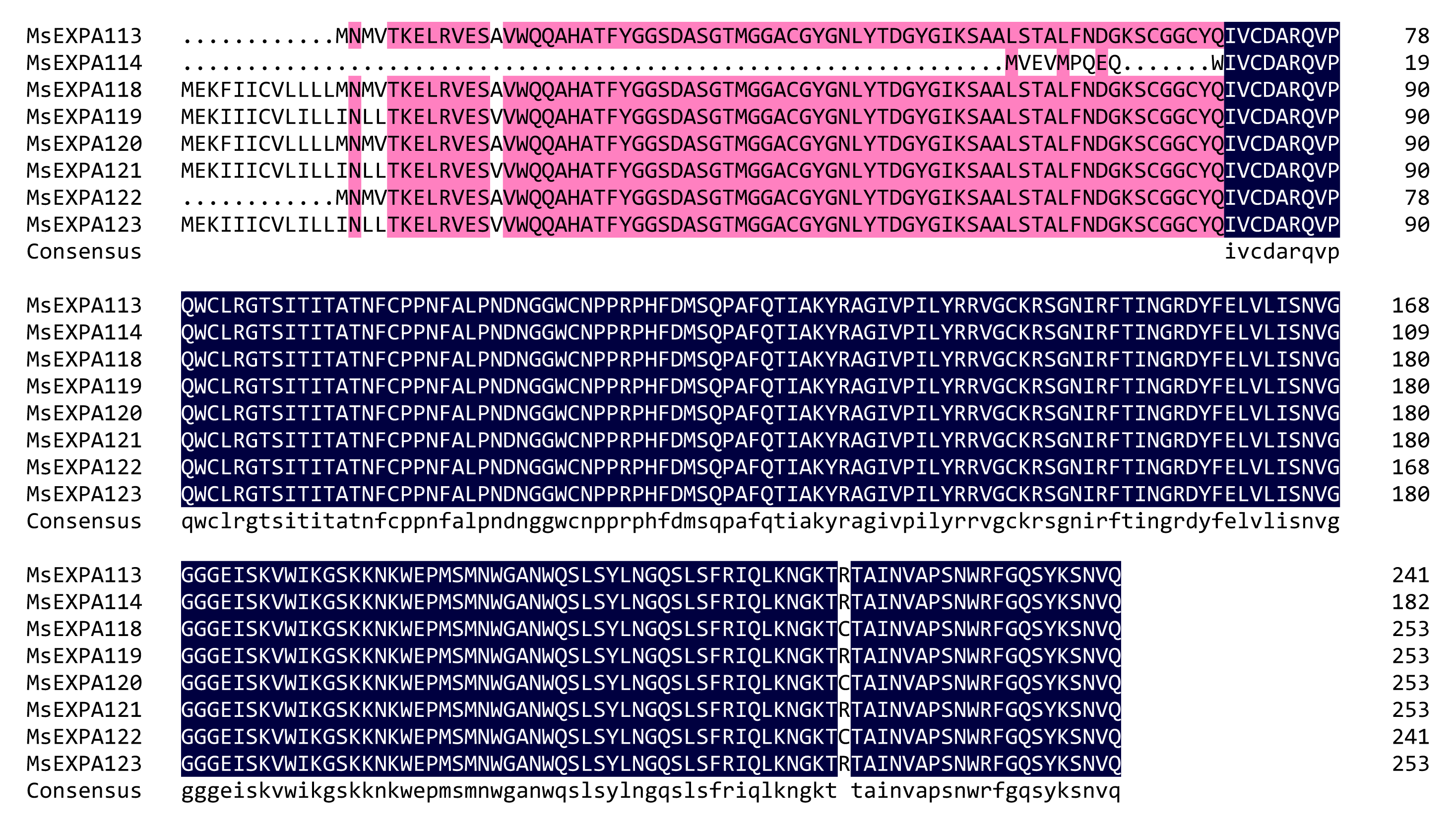

Supplement: Supplementary file 1 [file ijms-25-04700-s001.zip › Figure S1.tif]
